# Structurally Diverse Metabolites from the *Ophiorrhiza japonica* Bl. and Their Antioxidant Activities In Vitro and PPARα Agonistic Activities In Silico

**DOI:** 10.3390/molecules27165301

**Published:** 2022-08-19

**Authors:** Qing Bu, Yang Jin, Meng-Juan Xu, Lei Wu, Lin-Fu Liang

**Affiliations:** 1 College of Materials Science and Engineering, Central South University of Forestry and Technology, Changsha 410004, China; 2 School of Chinese Materia Medica, Nanjing University of Chinese Medicine, Nanjing 210023, China; 3 College of Forestry, Central South University of Forestry and Technology, Changsha 410004, China

**Keywords:** *Ophiorrhiza japonica*, ophiorrhizaquinone A, alkaloid, antioxidant, molecular docking

## Abstract

*Ophiorrhiza japonica* Bl. is a traditional Chinese materia medica widely used to treat several diseases. Chemical and pharmacological studies on *O. japonica* have been carried out; however, neither of them has been fully explored. In this study, an array of compounds was isolated from the title plant, including a new anthraquinone, ophiorrhizaquinone A (**1**), three alkaloids **2**–**4** and seven other compounds **5**–**11** with diverse structural types. Additionally, compounds **2**, **5**, **7**, **8**, **10** and **11** were isolated from the genus of *Ophiorrhiza* for the first time. Antioxidant bioassays in vitro using DPPH and ABTS were performed, and the results showed that compound **3** exhibited modest antioxidant activity with IC_50_ values of 0.0321 mg/mL and 0.0319 mg/mL, respectively. An in silico study of PPARα agonistic activities of compounds **2** and **3** was conducted by molecular docking experiments, revealing that both of them occupied the active site of PPARα via hydrogen bonds and hydrophobic interactions effectively. This study enriched both the phytochemical and pharmacological profiles of *O. japonica*.

## 1. Introduction

*Ophiorrhiza japonica* Bl., belonging to the Rubiaceae family, is widely distributed in Southern China [1]. Its whole plant is commonly referred to as “she gen cao” in traditional Chinese medicine (TCM), and is used to treat bronchitis, rheumatic arthralgia, injuries, irregular menstruation, etc. [2]. Interestingly, previous phytochemical studies of *O. japonica* revealed that it is composed of mainly alkaloids [3,4,5,6]. Recently, Liu’s group [3] disclosed two monoterpenoid indole alkaloids possessing a novel spirocyclic ring system, ophiorrhines A and B, from the title species. Their continued phytochemical research led to two undescribed alkaloids, ophiorrhines F and G, which are key biogenetic intermediates of ophiorrhines A and B [4]. To the best of our knowledge, there are only two pharmacological studies of the chemical constituents from *O. japonica*, both of which revealed the potent immunosuppressive activities [3,4]. However, neither phytochemical nor pharmacological profiles of *O. japonica* have been fully explored. Therefore, more studies on the isolation and identification of novel compounds with potent biological activities are regarded as necessary, as only a few compounds have been reported.

Dozens of research works revealed that the pathogenesis of various diseases, such as cancer, diabetes and neurodegeneration, are relevant to the damages caused by reactive oxygen species (ROS) [7]. Numerous therapeutic strategies were applied for treating ROS-related diseases, of which the antioxidants played important roles in various disciplines due to their abilities to eliminate ROS directly or to balance redox status in vivo [8]. Not surprisingly, the usage of natural antioxidants becomes an attractive topic, owing to the advantages of wide sources, strong activities and good affinities with biological surroundings [8]. It is found that some compounds from the genus *Ophiorrhiza* exhibited antioxidant activities [6]. However, no antioxidant bioassays were reported for *Ophiorrhiza japonica*. As reported, the family of peroxisome proliferator-activated receptors (PPARs), which can regulate the ROS levels over a wide range, from inhibition to induction, were the most prominent targets [9]. Their involvements in the regulation of ROS production and degradation underlie the therapeutic effects. Therefore, the PPARα was selected, and the agonistic activity was studied via molecular docking experiments.

Therefore, this study aimed to explore the phytochemical constituents of *O. japonica* and evaluate its antioxidant activity in vitro and PPARα agonistic activity in silico. To this purpose, the antioxidant potentials of the isolates were evaluated by DPPH (1,1-diphenyl-2-picrylhydrazyl) and ABTS (2,2′-azinobis-3-ethylbenzthiazoline-6-sulfonic acid) assays. Herein, the isolation, structure elucidation and preliminary biological studies in vitro and in silico are described.

## 2. Results and Discussion

**Figure 1:**
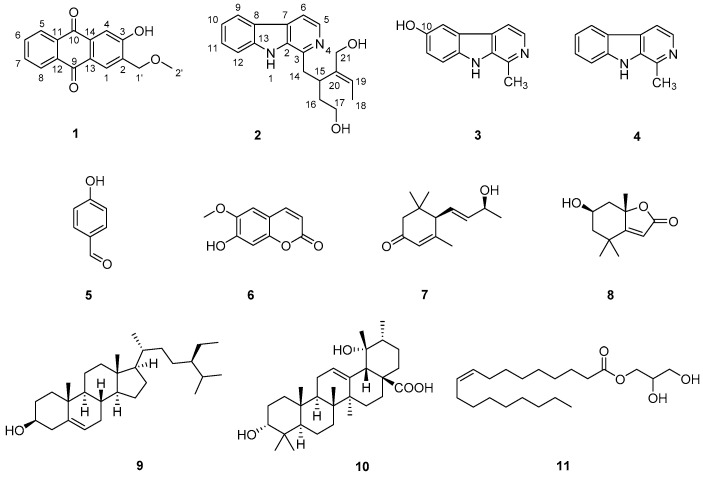
The chemical structures of compounds **1**–**11** obtained from *O. japonica*.

A phytochemical examination of the whole plant of *O. japonica* led to one new compound **1**, along with 10 known compounds **2**–**11** (Figure 1). Their chemical structures were identified by comparison of their NMR and optical rotation data with those in the literature.

Compound **1** was isolated as a yellow amorphous powder. Its molecular formula was established as C_16_H_12_O_4_ by a ion peak at *m*/*z* 267.0660 [M-H]^−^ (calcd. 267.0663 for C_16_H_1__1_O_4_) in the HREIMS spectrum (Figure S1), indicating 11 degrees of unsaturation. The IR absorption at 3358 and 1658 cm^−1^ suggested the presence of hydroxyl and carbonyl groups (Figure S8). The ^13^C NMR, DEPT, and HSQC spectra (Figures S3 and S4) of **1** revealed 16 carbon signals, including one oxygenated methyl (δ_C_ 59.1), one oxygenated sp^3^ methylene (δ_C_ 74.0), six sp^2^ methines (δ_C_ 134.2, 133.9, 128.1, 127.3, 127.3, and 114.9), and eight sp^2^ nonprotonated carbons (six carbons at δ_C_ 162.0, 136.5, 133.8, 133.8, 128.2, and 126.5, two ketonic carbonyls at δ_C_ 183.0 and 182.3) (Table 1). Six double bonds and two carbonyls accounted for eight of the eleven degrees of unsaturation; the remaining three degrees of unsaturation indicated that a tricyclic ring system belonged to the structure. Its ^1^H NMR data (Table 1, Figure S2) revealed that compound **1** consist of an ortho-substituted benzene ring (ring A) at δ_H_ 8.29 (2H, overlapped) and 7.78 (2H, overlapped), and a 1,2,4,5-tetrasubstituted benzene ring (ring C) at δ_H_ 8.02 (1H, s) and 7.72 (1H, s). Herein, a quinone ring (ring B) could be supposed from the left one ring and two ketonic carbonyls. The presence of a quinone ring was further supported by the mutiple absorption maxima between 200 and 400 nm (Figure S9). These spectroscopic features were similar to those of a known compound 3-hydroxy-1-methoxy-2-methoxymethylanthraquinone (**12**) [10].

**Figure 2:**
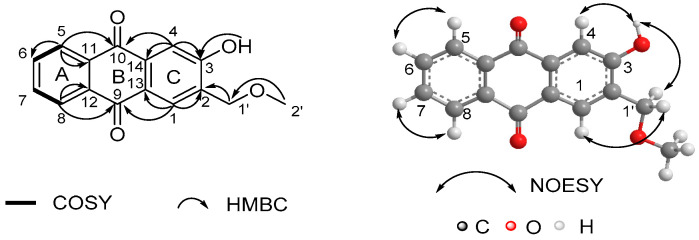
^1^H-^1^H COSY, key HMBC and NOESY correlations of compound **1**.

The only difference was at C-1 position, where the methoxyl functionality in **12** was absent in **1**. This replacement was consistent with their 30 mass units’ difference and evidenced by the HMBC correlations from H-1 (δ_H_ 8.02) to C-2 (δ_C_ 128.2), C-3 (δ_C_ 162.0), C-13 (δ_C_ 126.5) and C-14 (δ_C_ 136.5) (Figure S6). Due to the absence of the methoxyl group, the carbon chemical shift of C-1 (*δ*_C_ 128.1) was prominently shifted upfield (Δ*δ* ≈ +32 ppm) compared to **12**. Finally, the structure of **1** was unambiguously elucidated by ^1^H-^1^H COSY, HMBC and NOESY experiments (Figure 2, Figures S5–S7). The ortho-substituted benzene ring (ring A) in compound **1** could be deduced from the ^1^H-^1^H COSY correlations between the overlapped pairs of H-5/8 (δ_H_ 8.29) and H-6/7 (δ_H_ 7.78) and the HMBC correlations between the overlapped pairs of H-5/H-8 and C-11/C-12 (δ_C_ 133.8). The 1,2,4,5-tetrasubstituted benzene ring (ring C) was indicated by the HMBC correlations from H-1 (δ_H_ 8.02) to C-2 (δ_C_ 128.2), C-3 (δ_C_ 162.0), C-13 (δ_C_ 126.5) and C-14 (δ_C_ 136.5), and from H-4 (δ_H_ 7.72) to C-3 and C-14. The mutual HMBC correlations of H-1′ (δ_H_ 4.84)/C-2′ (δ_C_ 59.1) and H-2′ (δ_H_ 3.54)/C-1′ (δ_C_ 74.0) revealed the existence of a methoxymethyl chain. This side chain connected to ring C at C-2, indicating the diagnostic HMBC correlations from H-1′ to C-1 (δ_C_ 128.1), C-2 and C-3, which was further supported by the NOESY correlation of H-1′/H-1. A hydroxyl group was suggested at C-3 by the HMBC correlations from OH (δ_H_ 8.53) to C-2, C-3 and C-4 (δ_C_ 114.9), which was further supported by the NOESY correlation of OH/H-4. The characteristic HMBC correlations of H-5/C-11, H-5/C-10 (δ_C_ 183.0), H-8/C-12, H-8/C-9 (δ_C_ 182.3), H-4/C-10, H-4/C-14, H-1/C-9 and H-1/C-13 revealed the formation of a quinone ring (ring B). Thus, the structure of **1** was determined as shown in Figure 1.

The structures of 10 known compounds **2**–**11** were established by comparison of their NMR and optical rotation data with those in the literature, such as deppeaninol (**2**) [11], 6-hydroxyharman (**3**) [12], harman (**4**) [13], *p*-hydroxy benzaldehyde (**5**) [14], scopoletin (**6**) [15], 3-oxo-*α*-ionol (**7**) [16], loliolide (**8**) [17], *β*-sitosterol (**9**) [18], 3-*epi*-pomolic acid (**10**) [10] and 1-monoolein (**11**) [19], respectively. It may be worth pointing out that compounds **2**, **5**, **7**, **8**, **10** and **11** were isolated from the genus of *Ophiorrhiza* for the first time. Among them, **1** was an anthraquinone, **2**–**4** were β-carboline-type monoterpenoid indole alkaloids, **5** was a benzene derivative, **6** was a coumarin, **7** and **8** were norsesquiterpenes, **9** was a steroid, **10** was a ursane-type triterpene, and **11** was a lipid. This finding enriched the phytochemical profile of *O. japonica*.

All the isolated compounds from *O. japonica* were evaluated for their antioxidant activity by DPPH and ABTS assays. The results revealed that only compounds **2** and **3** exhibited radical scavenging abilities, respectively. Compound **2** exhibited moderate antioxidant activity with IC_50_ values of 2.70 mg/mL and 2.00 mg/mL, respectively. And compound **3** possessed the best antioxidant activity with IC_50_ values of 0.0321 mg/mL and 0.0319 mg/mL, while the positive control vitamin C had an IC_50_ value of 0.0154 mg/mL and 0.0164 mg/mL, respectively. By comparing the structures of the isolated alkaloids **3** and **4**, it seemed that the hydroxyl group at C-10 influenced the antioxidant activity, which donate a hydrogen atom to neutralize the free radicals, hence enhancing the oxidation potentials [20]. The comparison of **2** and **4** revealed that the more complex functionality at C-3 could help improve the antioxidant potency.

**Figure 3:**
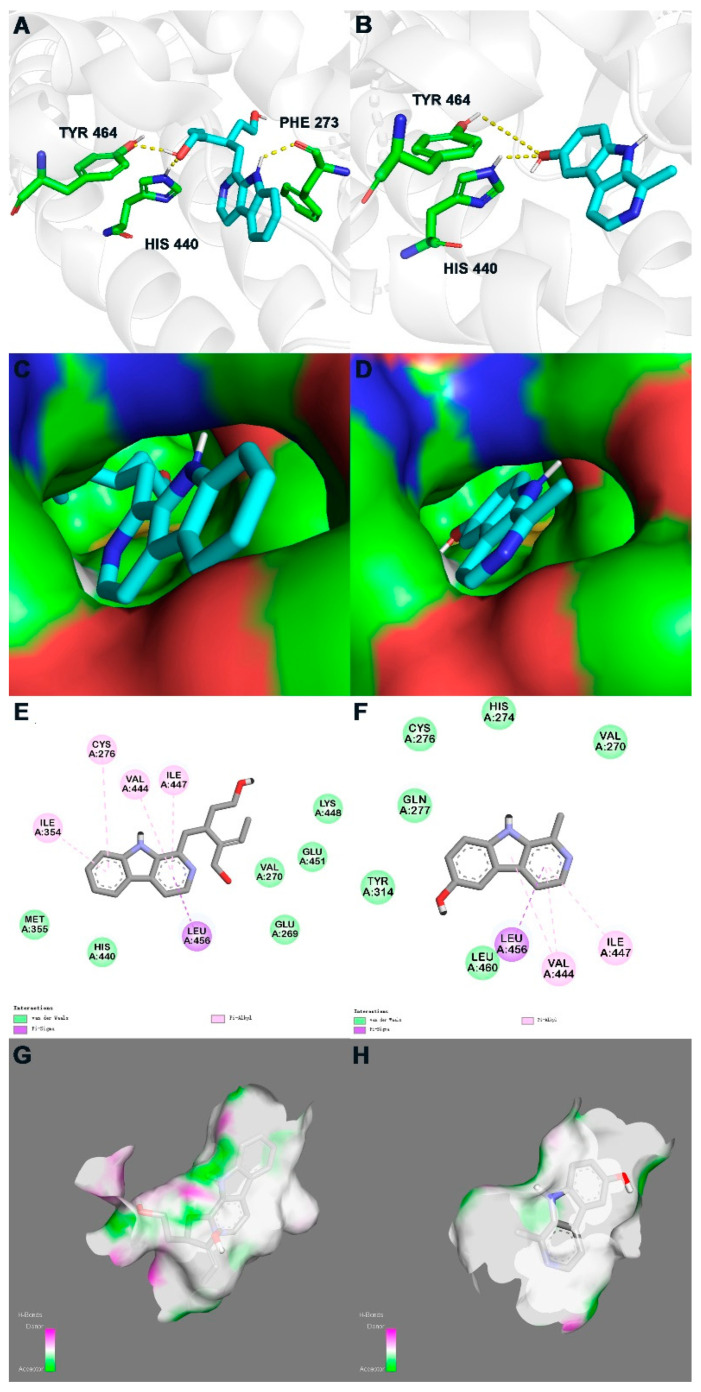
In silico binding mode of **2** and **3** at PPARα crystal structure 5HYK: first row—the transparent protein surface, in light grey color, and two compounds shown as sticks with atoms colored C cyan, N blue, O red, and H white, are shown to emphasize the clear combination of hydrogen bonds within the target pocket; second row—surfaces of 5HYK with combined compounds; third row—two-dimensional ligand interaction diagrams of two compounds at the PPARα domain; fourth row—receptor surface of H-bond. Left list (**A**,**C**,**E**,**G**) represents docking results of **2**; Right list (**B**,**D**,**F**,**H**) represents docking results of **3**.

The in silico study of PPARα agonistic activities of compounds **2** and **3** was conducted preliminarily by the molecular docking experiments, using the highly resolved PPARα crystal structure (PDB: 5HYK with a resolution of 1.83 Å). The molecular docking study revealed that compounds **2** and **3** occupied the same region as the ligand 65W, which was identified as a novel PPARα pan-agonist (also termed AL29-26). Figure 3 displays the binding modes of the isolated compounds with 5HYK.

For compound **3**, its C-10 hydroxy participated in hydrogen bonds with His440 and Tyr464, which were laying in the active site (Figure 3B,D). Discovery Studio Visualizer showed the pyrrole ring and pyridine ring of compound **3** occupied the hydrophobic pocket, which promoted π-σ and π-alkyl interactions with Val444, Ile447 and Leu456 (Figure 3F). For compound **2**, except for the same hydrogen bonds and hydrophobic interactions as compound **3**, H-1 and the benzene ring also participated in the interaction with Phe273, Cys276 and Ile354 (Figure 3A,C,E). The lower binding affinity of compound **2**, compared to that of compound **3** (Table 2), could be deemed to be related to the substituent at C-15 in an environment without an H-bonds donor or acceptor, which may reduce the binding stability with 5HYK (Figure 3G,H).

## 3. Materials and Methods

### 3.1. General Experimental Procedures

NMR spectra were measured on a Bruker DRX-500 or Bruker DRX-600 spectrometer (Bruker Biospin AG, Fällanden, Germany). The LREIMS and HREIMS data were recorded on a Finnigan-MAT-95 mass spectrometer (Finnigan-MAT, San Jose, CA, USA). Commercial silica gel (200–300 and 300–400 mesh, Qingdao Haiyang Chemical Group Co., Ltd., Qingdao, China), Sephadex LH-20 gel (Amersham Biosciences, Amersham, UK) were used for column chromatography, and precoated silica gel plates (GF-254, Yan Tai Zi Fu Chemical Group Co., Yantai, China) were used for analytical TLC. All solvents used for column chromatography were of analytical grade (Shanghai Chemical Reagents Co., Ltd., Shanghai, China)

### 3.2. Plant Material

*O. japonica* was collected from the hilly areas of Changning City, Hunan Province, China, and authenticated by Prof. Lei Wu of Central South University of Forestry and Technology (CSUFT). A botanical specimen (WL9962) was deposited at the herbarium of CSUFT.

### 3.3. Extraction and Isolation

The dried and powdered whole plant materials (1.0 kg) were extracted by maceration with ethanol (3 times, 7 days/time) at room temperature. The ethanol extract was evaporated under reduced pressure to give a dark residue. The ethanol extract was then suspended in water for liquid–liquid extraction and successively extracted with petroleum ether (P), ethyl acetate (E) and chloroform (C) to obtain their corresponding fractions.

The chloroform extract was subjected to silica gel column chromatography (200–300 mesh) by eluting with PE-EA at a ratio of 50:50 to 0:100 and obtained four fractions (Fr. C1–C4). Fr. C3 was purified by silica gel column chromatography (300–400 mesh, dichloromethane (D):methanol (M) 15:1) to yield compound **4** (45.9 mg). Fr. C4 was divided into two sub-fractions (Fr. C4A and C4B) by silica gel column chromatography eluted with D:M (25:1, 15:1). Fr. C4A was further purified by silica gel column chromatography (D:M 22:1) to afford compound **2** (49.7 mg). Similarly, compound **3** (18.7 mg) was obtained from Fr. C4B by chromatographing over silica gel eluted with D:M (10:1).

The ethyl acetate extract was fractioned by silica gel column chromatography, eluted with gradient P-E mixture (75:25 to 0:100) to afford five fractions (Fr. E1–E5). Compound **1** (3.7 mg) was obtained from Fr. E1 by chromatographing over silica gel eluted with 100 % D. Fr. E3 was purified by silica gel column chromatography (D:E 17:1) to yield compound **5** (8.7 mg). Two sub-fractions (Fr. E5A and E5B) were obtained from Fr. E5 by silica gel column chromatography eluted with D:M (10:1 to 0:1). Fr. E5A was further purified by silica gel column chromatography (P:1 1:1) to afford compound **6** (3.0 mg). Compounds **7** (4.1 mg) and **8** (9.7 mg) were obtained from Fr. E5B by chromatographing over Sephadex LH-20 gel eluted with P:D:M (2:1:1).

The petroleum ether extract was separated by silica gel column chromatography, eluted with gradient P-E mixture (100:0 to 0:100), yielding seven fractions (Fr. P1–P7). Fr. P5 was purified by silica gel column chromatography (P:E 8:1) to give compound **9** (48.1 mg). Similarly, compounds **10** (54.0 mg) and **11** (6.3 mg) were afforded from Fr. P5 and Fr. P6 by repeated silica gel column chromatography eluted with D:M (25:1 to 15:1) and D:M (16:1), respectively.

Ophiorrhizaquinone A (**1**): yellow amorphous powder; UV (MeOH) *λ*_max_ (log *ε*): 205 (0.439), 239 (0.303), 243 (0.301), 273 (0.585) nm; IR (KBr) *ν*_max_: 3443, 3358, 2922, 2851, 1658, 1632, 1583, 1572, 1344, 1122 cm^−1^; ^1^H (CDCl_3_, 600 MHz) and ^13^C (CDCl_3_, 125 MHz) NMR data, see Table 1; HRESIMS *m*/*z* 267.0600 [M − H]^−^ (calcd. for C_16_H_11_O_4_, 267.0663).

### 3.4. In Vitro Antioxidant Assays

The 1,1-diphenyl-2-picrylhydrazyl (DPPH) scavenging capability of the isolates was tested as described in [21]. As standard, vitamin C was used, with methanol as a blank. Similarly, the 2,2′-azino-bis-3-ethylbenzthiazoline-6-sulfonic acid (ABTS) scavenging capability of the isolates was tested as described in [22] using vitamin C for standard calibration. Ethanol was used as a blank. The percentage inhibition was deliberated using the formula below, and the results were expressed as IC_50_:

% inhibition = 100 × (Ac − As)/Ac

where Ac = absorption of the control, and As = absorption of the isolate.

### 3.5. Molecular Docking

The cocrystal structure of PPARα (PDB code 5HYK) was obtained from RCSB Protein Data Bank. The water molecules were removed in Autodock, while the binding site of compounds **2** and **3** on the PPARα receptor was consistent with that of 65 W. Moreover, the center of combined area was x = 8.938, y = 34.766, z = −17.821, spacing 0.375 (size x = 13.5; y = 13.5; z = 15.0). Using hydrogens and charges tool in edit, the receptor was prepared for docking. Ligands were sketched in ChemBioDraw program and uploaded to Autodock. Furthermore, ligands were obtained through ‘hydrogens’. The molecular docking module (Autodock Vina) was used for docking, and the simulation with the highest affinity energy which showed hydrogen bond interaction with the active site was analyzed and visualized in Pymol and Discovery Studio Visualizer.

## 4. Conclusions

In summary, a detailed chemical investigation of *O. japonica* led to the identification of an array of structurally diverse compounds, including one new anthraquinone (**1**), three β-carboline-type alkaloids (**2**–**4**), one benzene derivative (**5**), one coumarin (**6**), two norsesquiterpenes (**7** and **8**), one steroid (**9**), one ursane-type triterpene (**10**) and one lipid (**11**). All the isolates were evaluated for their antioxidant activities. Among them, compound **3** exhibited the best antioxidant capabilities in the DPPH and ABTS assays, which was similar to that of the positive control vitamin C. Preliminary analysis of the structures of the monoterpenoid indole alkaloids **2**–**4** revealed the hydroxyl group at C-10 and the substituent at C-3 could be crucial in this compound class for the enhancement of the antioxidant potency. With the assistance of molecular docking analysis, the binding modes of compounds **2** and **3** on PPARα were analyzed, revealing that both of them occupied the active site of PPARα via hydrogen bonds and hydrophobic interactions effectively. The discovery of these compounds of different types as well as their bioactive studies expanded the diversity of phytochemical and pharmacological profiles of *O. japonica*.

## Figures and Tables

**Table 1:** ^1^H NMR (δ_H_) and ^13^C NMR (δ_C_) data of compound **1** in CDCl_3_.

No.	δ_H_ (Mult.) ^a^	δ_C_^b^
1	8.02 (s)	128.1
2	-	128.2
3	-	162.0
4	7.72 (s)	114.9
5	8.29 (overlap)	127.3
6	7.78 (overlap)	133.9
7	7.78 (overlap)	134.2
8	8.29 (overlap)	127.3
9	-	182.3
10	-	183.0
11	-	133.8
12	-	133.8
13	-	126.5
14	-	136.5
1′	4.84 (s)	74.0
2′	3.54 (s)	59.1
3-OH	8.53 (s)	-

^a^ Recorded at 600 MHz. ^b^ Recorded at 125 MHz. Assignments were deduced by analysis of 1D and 2D NMR spectra.

**Table 2:** In silico molecular docking binding affinities of compounds **2** and **3** to PPARα crystal structure (PDB: 5HYK).

PPARα Crystal Structure	Compound ID	Affinity Energy (kcal mol^−1^)
5HYK	**2**	−6.9
	**3**	−7.9

## Data Availability

Not applicable.

## Supplementary Materials

The following supporting information can be downloaded at: https://www.mdpi.com/article/10.3390/molecules27165301/s1, Figure S1: HRESIMS spectrum of compound **1**; Figure S2: ^1^H NMR spectrum (600 MHz) of compound **1** in CDCl_3_; Figure S3: ^13^C NMR (BB + DEPT) spectrum (125 MHz) of compound **1** in CDCl_3_; Figure S4: HSQC spectrum (600 MHz) of compound **1** in CDCl_3_; Figure S5: ^1^H–^1^H COSY spectrum (600 MHz) of compound **1** in CDCl_3_; Figure S6: HMBC spectrum (600 MHz) of compound **1** in CDCl_3_; Figure S7: NOESY spectrum (600 MHz) of compound **1** in CDCl_3_; Figure S8: IR spectrum of compound **1**; Figure S9: UV spectrum of compound **1**.
