# Supplementary material for: Structurally Diverse Metabolites from the Ophiorrhiza japonica Bl. and Their Antioxidant Activities In Vitro and PPARα Agonistic Activities In Silico
Source: Molecules. 2022 Aug 19;27(16):5301. doi: 10.3390/molecules27165301 (PMC9412425; doi:10.3390/molecules27165301)
Supplement: Supplementary file 1 [file molecules-27-05301-s001.zip › molecules-1835088-supplementary.pdf]

Supplementary Materials

Structurally Diverse Metabolites from the *Ophiorrhiza japonica* Bl. and Their Antioxidant Activities In Vitro and PPARα Agonistic Activities In Silico

Qing Bu [1,†], Yang Jin [2,†], Meng-Juan Xu [1], Lei Wu [3] and Lin-Fu Liang [1,*]

[1] College of Materials Science and Engineering, Central South University of Forestry and Technology, Changsha 410004, China
[2] School of Chinese Materia Medica, Nanjing University of Chinese Medicine, Nanjing 210023, China
[3] College of Forestry, Central South University of Forestry and Technology, Changsha 410004, China
* Correspondence: lianglinfu@csuft.edu.cn
† These authors contributed equally to this work

Table of contents

Figure S1. HRESIMS spectrum of compound **1**
Figure S2. ^{1}H NMR spectrum (600 MHz) of compound **1** in $CDCl_3$
Figure S3. ^{13}C NMR (BB+DEPT) spectrum (125 MHz) of compound **1** in $CDCl_3$
Figure S4. HSQC spectrum (600 MHz) of compound **1** in $CDCl_3$
Figure S5. ^{1}H–^{1}H COSY spectrum (600 MHz) of compound **1** in $CDCl_3$
Figure S6. HMBC spectrum (600 MHz) of compound **1** in $CDCl_3$
Figure S7. NOESY spectrum (600 MHz) of compound **1** in $CDCl_3$
Figure S8. IR spectrum of compound **1**
Figure S9. UV spectrum of compound **1**

Qualitative Analysis Report

**Data Filename**	ESIH202202470-1.d	**Sample Name**	A8-A8-RE1
**Sample ID**		**Position**	P1-A4
**Instrument Name**	Agilent G6520 Q-TOF	**Acq Method**	20160324_MS_ESIH_NEG_1min.m
**Acquired Time**	7/25/2022 15:01:10	**IRM Calibration Status**	Success
**DA Method**	small molecular data analysis method.m	**Comment**	ESIH by fangsu

User Spectra

Fragmentor Voltage	Collision Energy	Ionization Mode
175	0	ESI

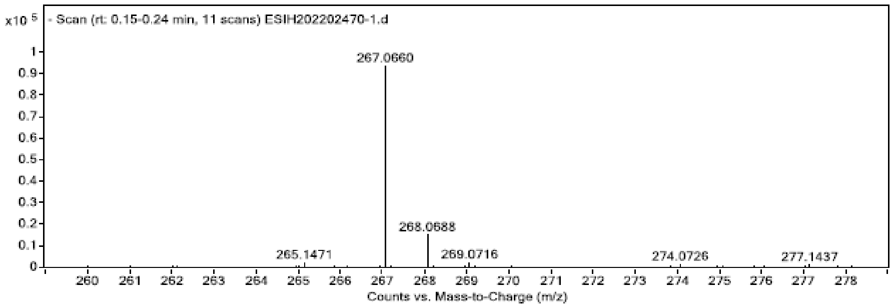

Formula Calculator Results

m/z	Calc m/z	Diff (mDa)	Diff (ppm)	Ion Formula	Ion
267.066	267.0663	0.33	1.22	C16 H11 O4	(M-H)-

--- End Of Report ---

Figure S1. HRESIMS spectrum of compound **1**

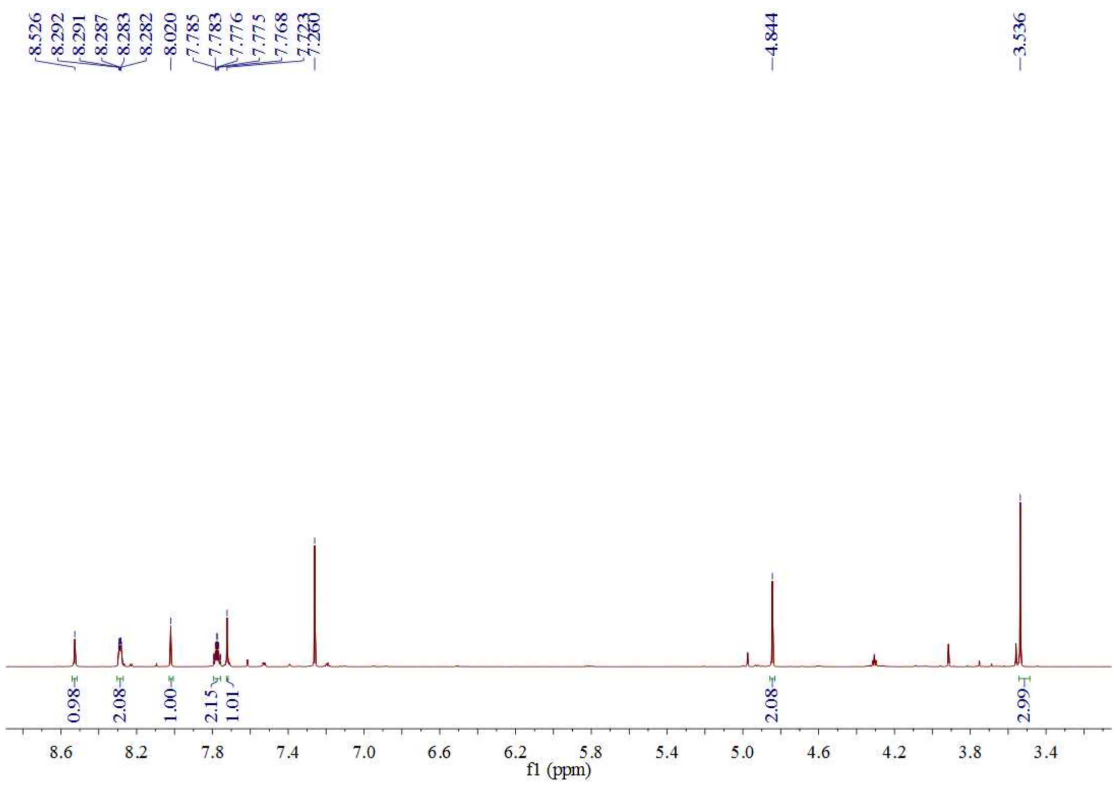

Figure S2. ^{1}H NMR spectrum (600 MHz) of compound **1** in $CDCl_3$

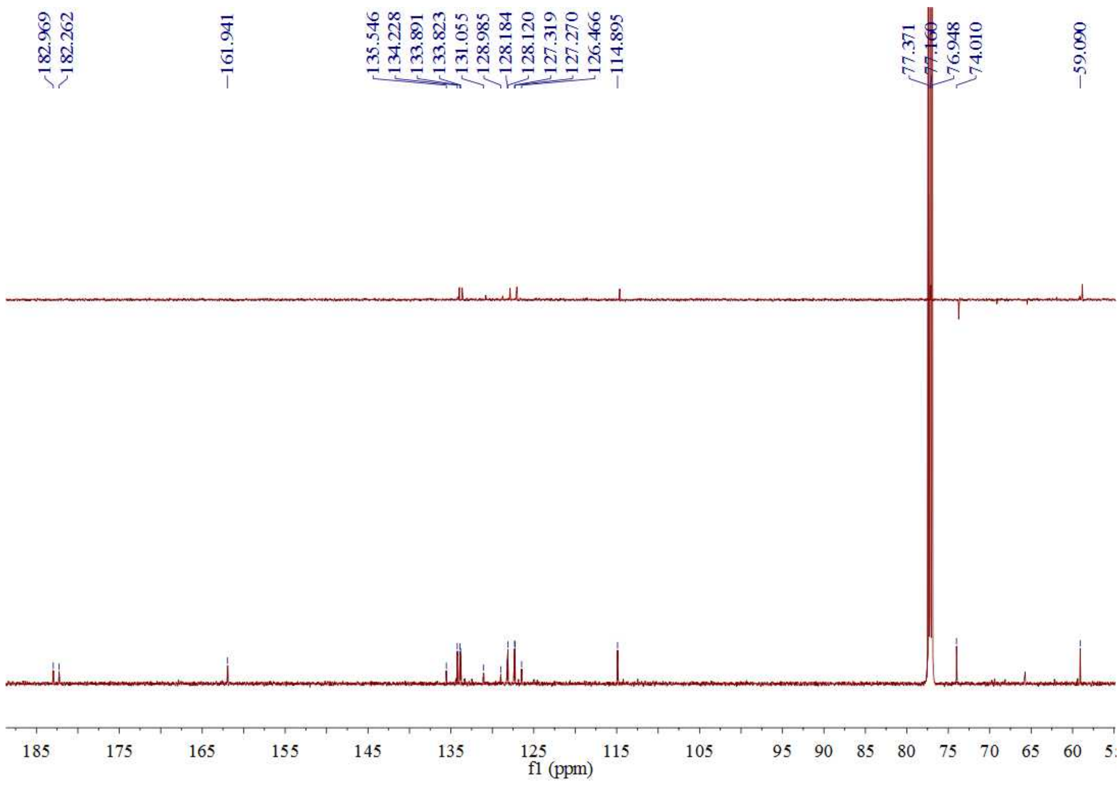

Figure S3. ^{13}C NMR (BB+DEPT) spectrum (125 MHz) of compound **1** in $CDCl_3$

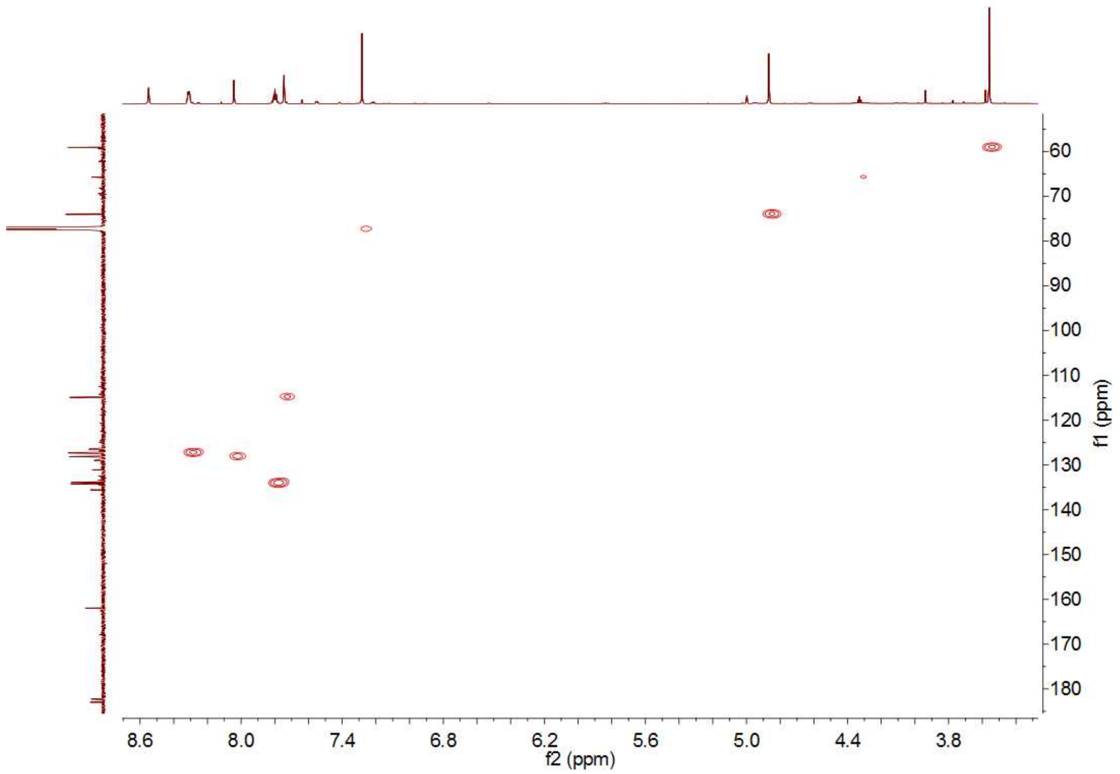

Figure S4. HSQC spectrum (600 MHz) of compound **1** in $CDCl_3$

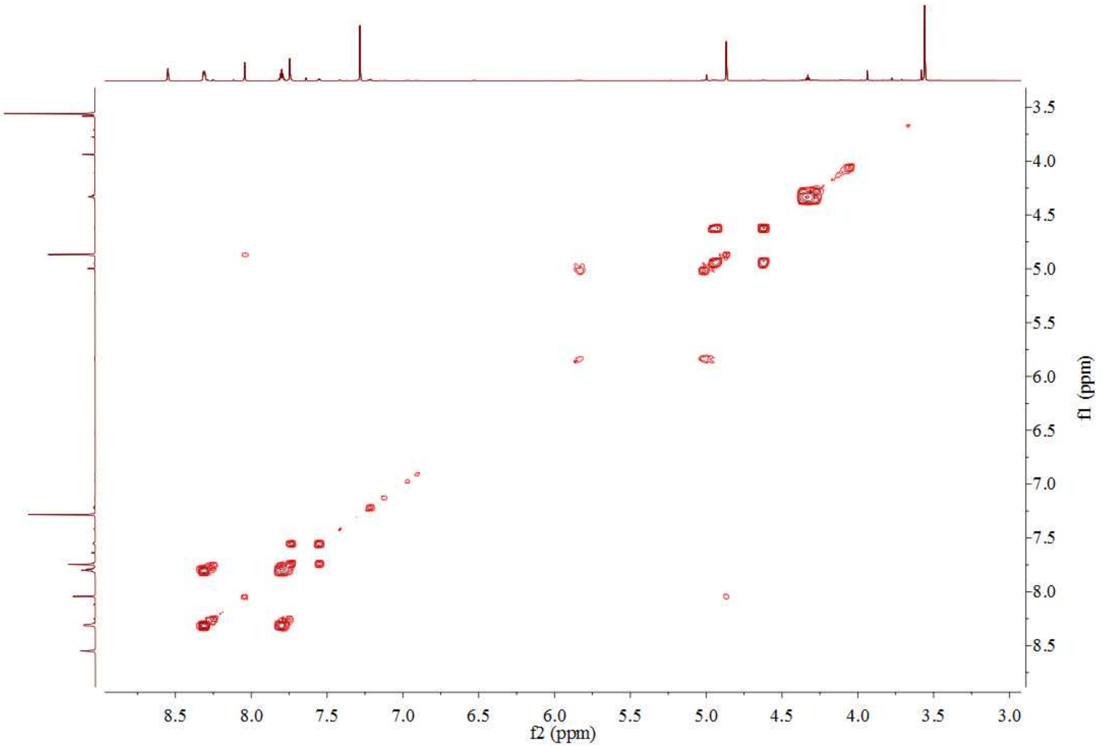

Figure S5. ^{1}H–^{1}H COSY spectrum (600 MHz) of compound **1** in $CDCl_3$

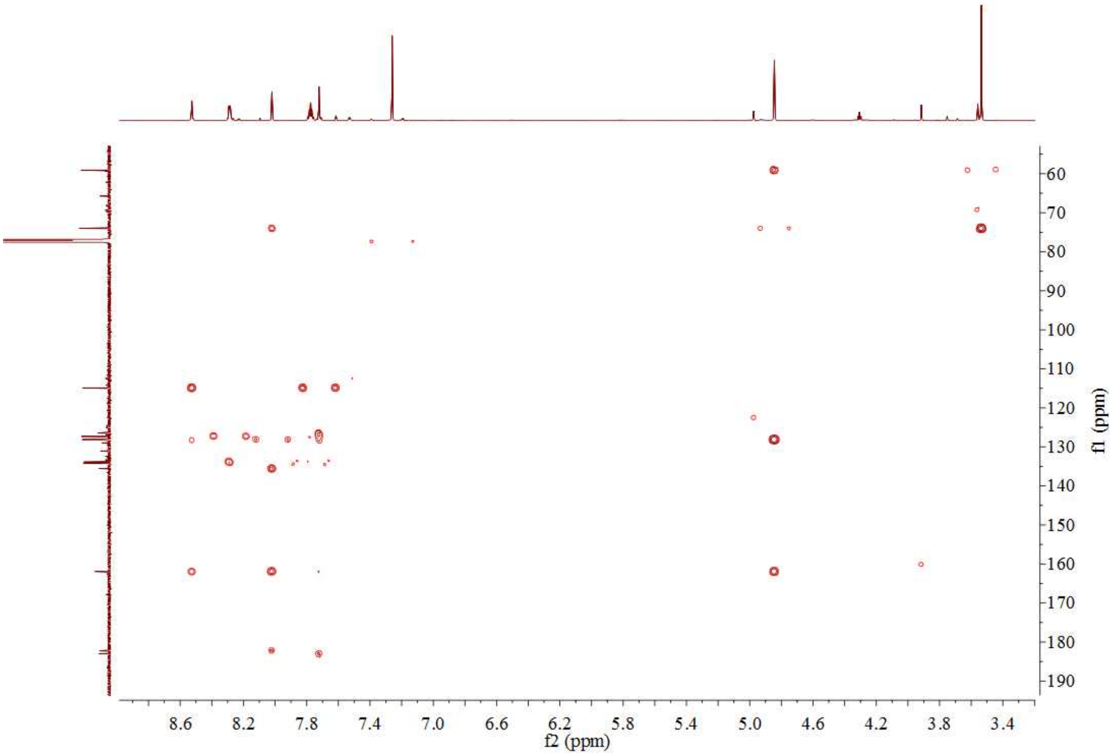

Figure S6. HMBC spectrum (600 MHz) of compound **1** in $CDCl_3$

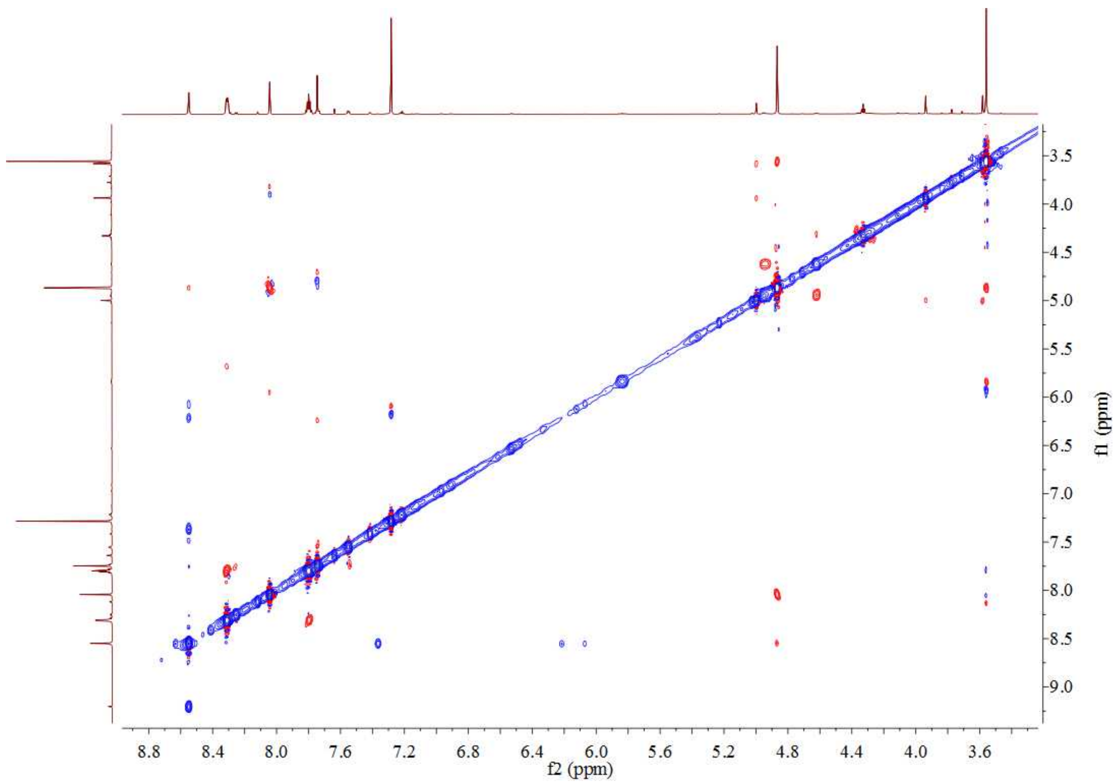

Figure S7. NOESY spectrum (600 MHz) of compound **1** in $CDCl_3$

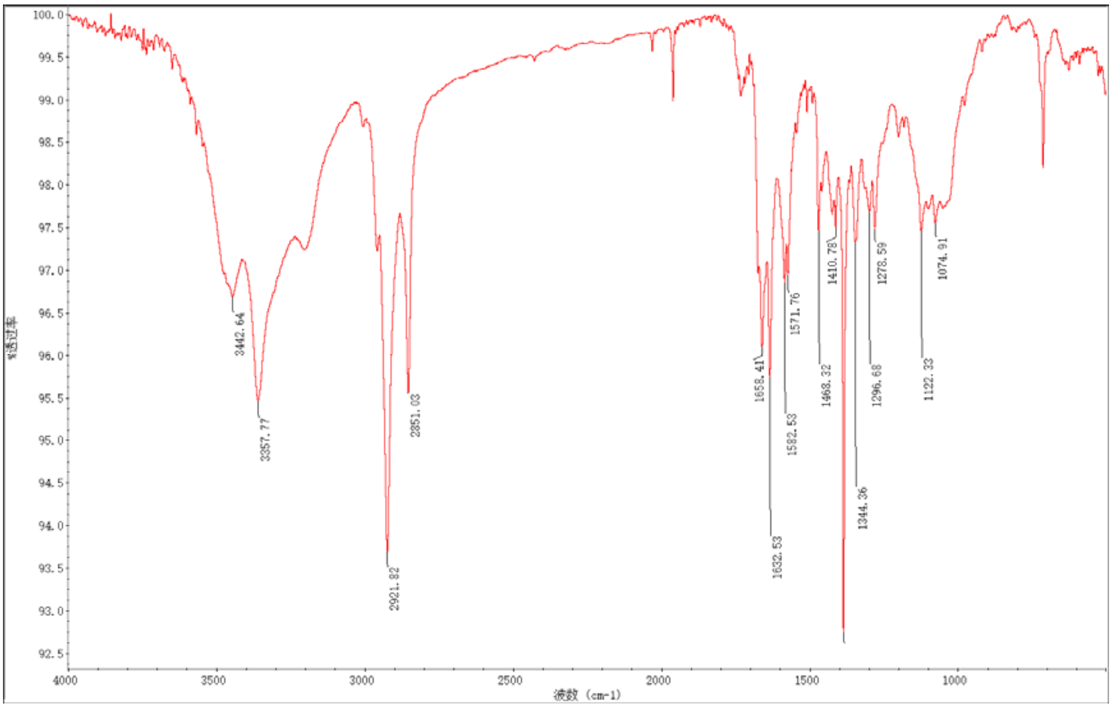

Figure S8. IR spectrum of compound **1**

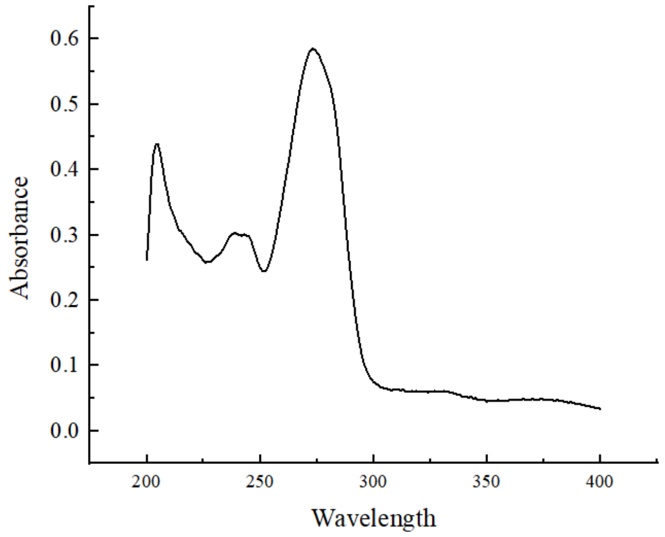

Figure S9. UV spectrum of compound **1**
